# A novel counterbalanced implementation study design: methodological description and application to implementation research

**DOI:** 10.1186/s13012-019-0896-0

**Published:** 2019-05-02

**Authors:** Mitchell N. Sarkies, Elizabeth H. Skinner, Kelly-Ann Bowles, Meg E. Morris, Cylie Williams, Lisa O’Brien, Anne Bardoel, Jenny Martin, Anne E. Holland, Leeanne Carey, Jennifer White, Terry P. Haines

**Affiliations:** 1 School of Primary and Allied Health Care, Monash University, Building G Peninsula Campus, McMahons Road, Frankston, Victoria 3199 Australia; 2 Allied Health Research Unit, Monash Health, 400 Warrigal Road, Cheltenham, Victoria 3092 Australia; 3 Department of Physiotherapy, Monash Health, 400 Warrigal Road, Cheltenham, Victoria 3092 Australia; 4 Department of Community Emergency Health and Paramedic Practice, Monash University, Building H Peninsula Campus, McMahons Road, Frankston, Victoria 3199 Australia; 5 La Trobe Centre for Sport and Exercise Medicine Research, La Trobe University, Bundoora, Victoria 3086 Australia; 6 North Eastern Rehabilitation Centre, Healthscope, Ivanhoe, Victoria 3079 Australia; 7 Peninsula Health, 4 Hastings Road, Frankston, Victoria 3199 Australia; 8 Department of Occupational Therapy, Monash University, Building G Peninsula Campus, McMahons Road, Frankston, Victoria 3199 Australia; 9 Department of Management and Marketing, Swinburne University, BA Buidling John Street, Hawthorn Campus, Hawthorn, Victoria 3122 Australia; 10 Swinburne University, John Street, Hawthorn, Victoria 3122 Australia; 11 Alfred Health and La Trobe University, 99 Commercial Road, Melbourne, Victoria 3004 Australia; 12 Occupational Therapy, School of Allied Health, La Trobe University, Bundoora, Victoria 3086 Australia; 13 Neurorehabilitation and Recovery, Melbourne Brain Centre, Florey Institute of Neuroscience and Mental Health, 245 Burgundy Street, Heidelberg, Victoria 3084 Australia

**Keywords:** Crossover, Counterbalanced, Method, Design, Research, Implementation, Context, Strategy, Randomised controlled trial, Study

## Abstract

**Background:**

Implementation research is increasingly being recognised for optimising the outcomes of clinical practice. Frequently, the benefits of new evidence are not implemented due to the difficulties applying traditional research methodologies to implementation settings. Randomised controlled trials are not always practical for the implementation phase of knowledge transfer, as differences between individual and organisational readiness for change combined with small sample sizes can lead to imbalances in factors that impede or facilitate change between intervention and control groups. Within-cluster repeated measure designs could control for variance between intervention and control groups by allowing the same clusters to receive a sequence of conditions. Although in implementation settings, they can contaminate the intervention and control groups after the initial exposure to interventions. We propose the novel application of counterbalanced design to implementation research where repeated measures are employed through crossover, but contamination is averted by counterbalancing different health contexts in which to test the implementation strategy.

**Methods:**

In a counterbalanced implementation study, the implementation strategy (independent variable) has two or more levels evaluated across an equivalent number of health contexts (e.g. community-acquired pneumonia and nutrition for critically ill patients) using the same outcome (dependent variable). This design limits each cluster to one distinct strategy related to one specific context, and therefore does not overburden any cluster to more than one focussed implementation strategy for a particular outcome, and provides a ready-made control comparison, holding fixed. The different levels of the independent variable can be delivered concurrently because each level uses a different health context within each cluster to avoid the effect of treatment contamination from exposure to the intervention or control condition.

**Results:**

An example application of the counterbalanced implementation design is presented in a hypothetical study to demonstrate the comparison of ‘video-based’ and ‘written-based’ evidence summary research implementation strategies for changing clinical practice in community-acquired pneumonia and nutrition in critically ill patient health contexts.

**Conclusion:**

A counterbalanced implementation study design provides a promising model for concurrently investigating the success of research implementation strategies across multiple health context areas such as community-acquired pneumonia and nutrition for critically ill patients.

**Electronic supplementary material:**

The online version of this article (10.1186/s13012-019-0896-0) contains supplementary material, which is available to authorized users.

## Background

The movement to translate research evidence into healthcare policy and practice is well established [1–3]. Delays in the uptake of research evidence can prolong the provision of ineffective, low-value, and even potentially harmful healthcare interventions [4, 5]. In response, governments, organisations, and health professionals are increasingly expected to ensure policy, and practice is informed by high-quality, contemporaneous research. Implementation research has been promoted as one way to facilitate the translation of research into practice [6]. This developing field of research evaluates the success of strategies such as knowledge brokering [7, 8], algorithms [9], and multifaceted approaches [10, 11] for individual and organisational change. Health service researchers have increasingly recognised implementation research as a field of science [12], although the benefits of many implementation attempts remain unclear [7, 13].

If the translation of research into practice is to be improved, it is important to understand which implementation strategies are effective and cost-effective. One approach is to compare one group receiving a strategy against another receiving usual care control conditions. However, often there is not only interest in whether a strategy is effective, but also in whether one strategy is more or less effective than another. Some strategies with minimal success may still be efficient if they are also low-cost. Likewise, the cost of successful strategies must be considered when making implementation resource allocation decisions [14]. In addition, many healthcare interventions found to be efficacious in one health context fail to translate benefits to effective patient care outcomes when implemented across diverse contexts [15]. Therefore, comparative effectiveness study designs that compare multiple strategies across different contexts are required.

Parallel cluster randomised controlled trials are commonly used in implementation science. These designs are amenable to evaluating studies of implementation when practice change is desired on a service level rather than an individual level [16]. However, many potential difficulties exist in the application of traditional research methodologies such as parallel designs to studies of implementation success. Logistical challenges in recruitment, implementation strategy delivery, and ensuring adequate statistic power in analyses have been reported in randomised controlled trials that did not reach recruitment targets [17]. In addition, some organisations and individuals are more ready for change than others due to the differences in organisational culture, previous history with change, and differences in individuals’ resistance to change [18, 19]. When these organisational and individual differences are combined with small sample sizes, this can lead to imbalances in factors that impede or facilitate change between intervention and control groups. Performing post hoc analysis and measuring additional factors such as ‘organisational readiness for change’ have historically been used to account for these issues [20–22]. However, doing so creates additional data collection burden and may still not adequately control for the differences between organisations, as these measures have been found to involve conceptual ambiguities and limited evidence of reliability or validity [23]. It would be preferable to mitigate this potential source of bias and minimise data collection burden through a design-based solution [24].

One approach to minimise inter-group variation is to use within-cluster repeated measure designs. These differ from between-cluster designs by exposing each cluster to the strategies being evaluated and comparing the effect within clusters rather than between [25]. Crossover studies are a commonly used within-cluster design, which provides each cluster with a random sequence of strategies to counterbalance order effects in repeated measure designs. The limitation of crossover designs in this setting is that exposure to an implementation strategy can permanently contaminate study clusters, so their characteristics are no longer comparable to what they were before they were exposed to the alternate condition. We propose a novel application of counterbalanced design to implementation science, which allows the concurrent comparison of interventions while minimising inter-group differences and the risk of contamination. In this paper, we firstly discuss the rationale and implications of using such an approach. We then describe a hypothetical example application of this approach to compare the effectiveness of two implementation strategies involving a ‘video-based’ evidence summary and ‘written-based’ evidence summary.

## Methods

### Common randomised study designs used in implementation research

**Fig. 1:**
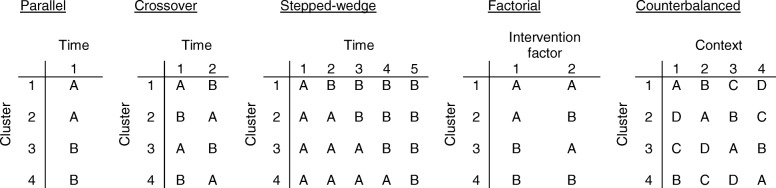
Contrast in condition allocation between commonly used randomised study designs in implementation research

The more traditional cluster randomised study design used in implementation research is a between-cluster parallel design. As parallel designs rely on comparing different clusters response to study conditions over the same time period, there is a risk of imbalance between the study groups where small sample sizes are recruited. Alternatively, researchers may consider within-cluster designs, such as crossover and stepped-wedge studies. These designs randomly assign clusters to a sequence of study conditions over a series of time periods. However, there is a risk that exposure to one implementation strategy will contaminate the cluster, so they are no longer comparable in these designs. Factorial designs allow clusters to experience all combinations of conditions, where there are two or more ‘factors’, each with different ‘levels’. One of the advantages is the ability to look at interaction effects between the factors. However, this design may also contaminate clusters after initial exposure to implementation strategies. In the proposed counterbalanced implementation study, units are randomised to alternative interventions; the different levels are applied in different contexts to reduce the risk of contamination. Figure 1 contrasts the counterbalanced design with other commonly used designs in implementation research.

### The counterbalanced implementation study methodological design

This design differs from conventional studies in that the implementation strategy (independent variable) can take on two or more levels which are evaluated across an equivalent number of health contexts (e.g. inpatients, outpatients, medical, surgical), using the same outcome (dependent variable). This design limits each cluster to one distinct strategy related to one specific context, and therefore does not overburden any cluster to more than one focussed implementation strategy for a particular outcome, and provides a ready-made control comparison, holding fixed. Unlike conventional designs, the different levels of the implementation strategy (independent variable) do not need to be provided sequentially; they can be provided concurrently, thereby eliminating contamination and period effects. Each level of the implementation strategy (independent variable) being delivered uses a different health context within each individual cluster to avoid contamination. This approach is represented most simply by a study comparing two implementation strategies (i.e. one independent variable that has two levels):

**Table:** 

R	XA1|B1	XA2|B2	O
R	XA2|B1	XA1|B2	O

Where R indicates the random assignment of units to conditions, O represents the unit outcome assessment, A is the implementation strategy, and B is the health context.

**Fig. 2:**
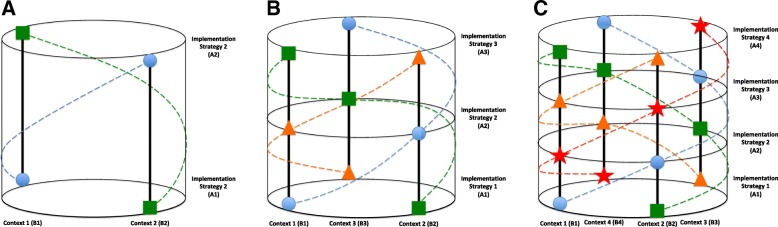
**a**–**c** Counterbalanced implementation study model. Circle, participant group 1; square, participant group 2; triangle, participant group 3; star: participant group 4

In this model, the XA1|B1 and XA2|B2 study group receives implementation strategy 1 (e.g. audit and feedback) in health context 1 (e.g. surgical inpatients) and strategy 2 (e.g. knowledge broker) in context 2 (e.g. medical outpatients), whereas the XA2|B1 and XA1|B2 study group receives implementation strategy 2 (e.g. knowledge broker) in health context 1 (e.g. surgical inpatients) and strategy 1 (e.g. audit and feedback) in context 2 (e.g. medical outpatients), see Fig. 2a for a three-dimensional representation.

The counterbalanced approach is not limited to two-level applications. Theoretically, a factorial approach could be used for any number of the implementation strategy and health context combinations. However, there may be a risk of imbalance in characteristics between the clusters, particularly with low recruitment rates. Therefore, any number of implementation strategies can be examined; however, a new health context would ideally be added for each additional implementation strategy included. This process is illustrated with the three-level helical study model:

**Table:** 

R	XA1|B1	XA2|B2	XA3|B3	O
R	XA2|B1	XA3|B2	XA1|B3	O
R	XA3|B1	XA1|B2	XA2|B3	O

In this model, the XA1|B1, XA2|B2, and XA3|B3 study group receive implementation strategy 1 in health context 1, strategy 2 in context 2, and strategy 3 in context 3. The XA2|B1, XA3|B2, and XA1|B3 study group receives implementation strategy 2 in health context 1, strategy 3 in context 2, and strategy 1 in context 3, and similarly for the XA3|B1, XA1|B2, and XA2|B3 study group, see Fig. 2b for a three-dimensional representation. The described three-sequence trial has six potential choices of assignment. In order to reduce the potential risk of order effects for both concurrent and sequential study condition assignment, a randomly selected balanced design could be achieved by matched randomisation of the three different strategies and contexts with blocks. Figure 2c illustrates three dimensionally how a four-level study model can be applied. The advantage of this model is systematic replication without contamination.

We next illustrate a hypothetical counterbalanced implementation study example. This will demonstrate the pragmatic comparison of ‘video-based’ and ‘written-based’ evidence summary research implementation strategies for changing clinical practice in community-acquired pneumonia and nutrition in critically ill patient health contexts. For simplicity, a two-level counterbalanced implementation study is presented, with the effectiveness of two different implementation strategies examined in two different health contexts.

## Results

### Hypothetical counterbalanced implementation study example

Two potential priority health contexts for the implementation of research evidence in practice are ‘management of community-acquired pneumonia’ and ‘nutrition in critically ill patients’. Community-acquired pneumonia is the second highest cause of mortality globally [26]. Evidence suggests that corticosteroid therapy may reduce mortality, need for mechanical ventilation, hospital length of stay, time to clinical stability, and severe complications [27–29]. Despite these findings, poor adherence to guidelines for the use of corticosteroids to treat community-acquired pneumonia has been reported [9, 30]. A second health context where current practice does not always align with the most contemporaneous research is nutrition in critically ill patients. Despite guidelines recommending the provision of early enteral nutrition for critically ill patients [31–35], many do not receive adequate nutritional support [34–36].

Two potential strategies which could be used to implement research into practice in the management of community-acquired pneumonia and nutrition in critically ill patient health contexts are ‘video-based’ research evidence summaries and ‘written-based’ research evidence summaries. Written-based evidence summaries have been a conventional method for evidence dissemination through scientific journal articles, abstracts, books, and editorials [13, 37]. There has been some examination of the effectiveness of written-based evidence summaries [38, 39]. However, this evidence is limited and does not include comparison with a non-written evidence summary approach. Similarly, video-based evidence summaries have been examined in some areas such as falls prevention education [40]. However, the results may not be applicable to other health contexts.

A counterbalanced implementation study could examine the success of written (A1) versus video (A2) evidence summaries for translating research into practice for community-acquired pneumonia (B1) and nutrition in critically ill patients (B2). This study model would be conducted as follows:

**Table:** 

R	XA1|B1	XA2|B2	O
R	XA2|B1	XA1|B2	O

**Fig. 3:**
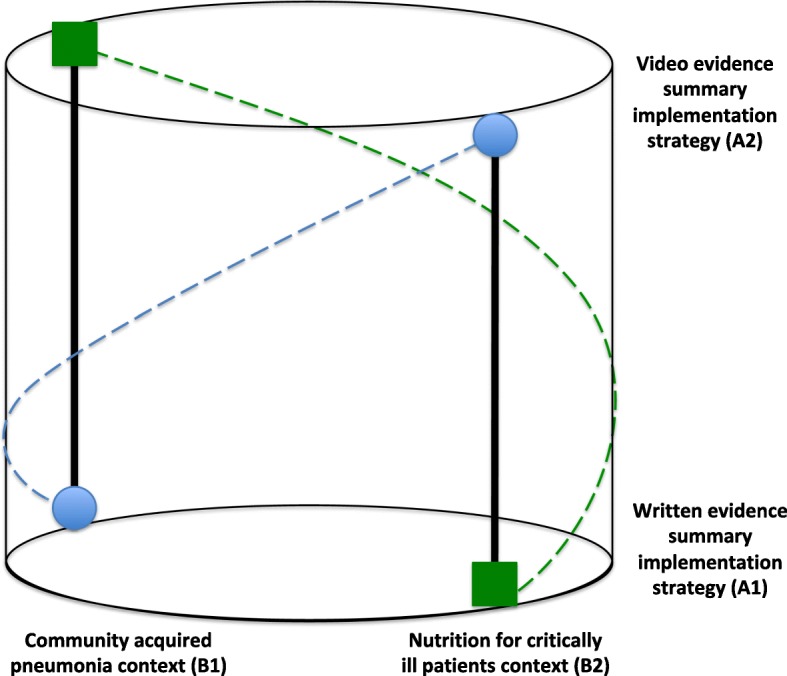
Two-level counterbalanced study model for written and video evidence summaries in community-acquired pneumonia and nutrition for critically ill patients

In this model, the XA1|B1 and XA2|B2 study group receives the written-based evidence summary implementation strategy (A1) in community-acquired pneumonia (B1) and the video-based evidence summary implementation strategy (A2) in nutrition for critically ill patient contexts (B2). The XA2|B1 and XA1B2 study group would receive the opposite, video-based evidence summary implementation strategy (A2) in the community-acquired pneumonia context (B1) and the written-based evidence summary implementation strategy (A1) in the nutrition for critically ill patient context (B2), see Fig. 3 for a three-dimensional representation.

### Measuring success in a counterbalanced implementation study

How to best measure implementation success is a developing concept in this field of science [7, 41]. Researchers must clearly define their aims before planning outcome measurement, as process measures may not necessarily translate to changed behaviour or improved patient outcomes, and vice versa. Potential outcomes can be conceptualised as an educational intervention measured according to the four-level Kirkpatrick model hierarchy: (1) reaction, (2) learning, (3) behaviour, and (4) results [42]. In addition, a number of process measures and implementation-specific outcomes can be considered [41]. Arguably, results such as patient and health service outcomes should be the standard for judging the success of an implementation strategy. However, it is important to understand the mechanisms leading to those changes and the sustainability of change.

Several logistical difficulties in the measurement of patient or organisation outcomes are posed in the counterbalanced implementation study paradigm. As multiple health contexts are considered, several different context-specific outcomes may need to be measured. In the hypothetical example, the time required to achieve 80% of the calculated energy intake goal may be an appropriate measure for the nutrition in critically ill patients example [32]; however, for community-acquired pneumonia, pneumonia-associated complications may be an appropriate measure [9]. Data would also need to be clustered for analysis, which potentially limits the statistical power if the implementation strategies were delivered at the ward or hospital level (Additional file 1). Another consideration is whether recruitment rates would reach an adequate threshold so that the number of health professionals in each cluster is not sub-therapeutic within the cluster. For example, a video- or written-based evidence summary aiming to improve corticosteroid use in the management of community-acquired pneumonia would need to recruit enough medical, nursing, and allied health staff on each ward to be able to change the outcomes at the aggregate ward cluster level. This problem could be offset by requiring a smaller overall sample size from which to collect outcomes, as balanced within-cluster repeated measure study designs may require a relatively smaller sample size than other unbalanced designs to achieve adequate statistical power [43].

Implementation-specific outcomes and changes in attitudes, learning, and behaviour may provide a more pragmatic, adequate measure of success in a counterbalanced implementation study, if assumptions about flow-on effects from process measures to health outcome changes are avoided and setting-specific organisational factors are considered. Whichever level of outcome from the Kirkpatrick hierarchy is selected to be the primary outcome in a counterbalanced trial, it is important to consider using the same outcome either within or across health contexts depending on the focus of the study, as this may allow pooling of results across implementation studies. Where a health outcome is the chosen primary outcome measure, a generic scale (such as a generic health-related quality of life scale) could also be applied within or across health contexts to allow comparison of success across studies. It is worth noting that generic scales can be less responsive than disease-specific scales, which may have implications for the statistical power and sample size of the study [44].

### Statistical analysis considerations

Analysis of a counterbalanced implementation study for the effectiveness of implementation strategies in each context should follow intention-to-treat conventions based on paired data, given participants act as their own controls [45]. Both superiority and non-inferiority analytical tests can be performed depending on the focus of the study and outcome measures of interest.

The overall effect of an implementation strategy can be derived using a single mixed effects generalised linear model (Additional file 2). In this statistical model, each cluster (unit of randomisation) is treated as a random effect (nesting where relevant), and each implementation strategy and (separately) context as a fixed effect (to account for the potential for differential exposure of implementation strategies to different context areas through non-permuted randomisation). Clusters are considered to be uncorrelated across health contexts. A two-level model is presented, however, multilevel approaches can be applied.

Interaction effects also may need to be considered in a counterbalanced implementation study (Additional file 3). This is because an implementation strategy may be more effective in one health context than another. Including an interaction effect term in the statistical model can be used to further clarify the relationships between dependent and independent variables. The model presented in Additional file 3 includes the same statistical model described previously with the addition of an interaction effect term.

For the analysis of counterbalanced designs in implementation science, consideration of whether health context is treated as a fixed or random factor is needed. An implementation study could also be designed with the health context as a random factor, providing an efficient way of producing generalisable knowledge. Mixed effects modelling with multiple crossed random effects have been used to evaluate similar designs in social science research [46–48]. This research often counterbalances participant response to test items under different conditions or treatment response for different stimuli. In this setting, both participants and condition/stimuli are considered random factors, as they have been sampled from a larger population. The inference is that researchers can generalise the findings across participants and condition/stimuli.

### Sample size and power calculations

For this study design, sample size estimations can be calculated in two steps. First, a power analysis for a two-sampled paired-proportions test is conducted assuming two independent samples to determine the sample size required for a between-cluster study design. This calculation is then followed by an adjustment for the relative efficiency (RE) of within-cluster designs. In order to illustrate this two-stage approach, an example sample size and a power calculation are presented.

For a study with three levels of context and implementation strategy, a sample size of 50 clusters (unit of randomisation) per group (150 clusters total) would provide 80% power at a 0.05 significance level with a two-sample paired-proportions test adjusted for the relative efficiency of within-cluster designs examining a pairwise contrast between two levels of implementation strategy across all three contexts, under the assumption that 60% of those in an intervention group align with the desired practice change and 40% in the control group. This process begins with a sample size of 107 clusters (unit of randomisation) across 3 levels of context and implementation strategy levels, generating 321 cluster-level outcome measurement observations. In this first step, a power analysis for a two-sample paired-proportions test examining a pairwise contrast between two levels of implementation strategy across all three contexts would provide 80% power at a 0.05 significance level, under the assumption that 60% of those in an intervention group align with the desired practice change and 40% in the control group. In the second step, adjustment for the relative efficiency (RE) of repeated measures in a counterbalanced design can be performed using the formula: RE = 0.5[(1 − Pc − *p*)/(1 + (*n* − 1)*p*)], where *n* is the number of units within each cluster across periods, *p* is the intra-class correlation coefficient (ICC) between units in the same cluster at the same time point, and Pc is the the inter-period correlation [49, 50]. One unit within each cluster across three periods, an assumed ICC of 0.001 between units and an inter-period correlation of 0.0442, leads to an estimated relative efficiency for crossover in the counterbalanced design of 0.4685. Once the between-cluster sample size is multiplied by the relative efficiency of within-cluster design (107 × 0.4685), this adjustment creates the target study sample size of 50 clusters (unit of randomisation) per group (150 total clusters total).

### Planning a counterbalanced implementation trial design

Implementation studies can be designed to change practice at the organisational level (e.g. health services), departmental level (e.g. hospital wards), health professional level (e.g. medical staff or physiotherapists), or patient level (e.g. inpatient or outpatients). Selecting the unit level of analysis is dependent on the focus of the study, and researchers also need to consider the feasibility of recruitment, implementation strategy delivery, and statistical power. Wilson and colleagues reflected on a randomised controlled trial attempt which did not reach recruitment targets, reporting that recruiting at a departmental or divisional unit of allocation, rather than at the level of individual staff members, may provide more meaningful analysis of implementation success [17]. However, this approach has limitations in statistical power, which would need to be addressed.

Planning a counterbalanced implementation study involves the selection of both health contexts and implementation strategies. We recommend that researchers engage in a consultative process with potential participant organisations and individuals, which, in turn, may improve the recruitment rates and fidelity of interventions. This type of early engagement is important because it can help to ensure health contexts and implementation strategies are relevant to participating individuals and organisations. Consultation would identify the context areas of interest to patients, clinicians, managers, policy-makers, and organisations. A prioritisation process may then provide a mechanism for selecting the specific health contexts for implementation in a counterbalanced study. Limitations could be imposed on the number of helices (strategy/context combinations) in the study, or several different studies could be run. Health contexts should involve interventions, assessments, policies, programmes, or practices with good levels of evidence to support both effectiveness and cost-effectiveness prior to implementation.

We recommend the selection of implementation strategies for examination that involve the application of an evidence-based framework, model, or theory [51]. Evaluation of implementation strategies can be aimed at describing the process of translating research into practice, understanding different variables which influence implementation outcomes, or examining strategy success depending on the overall aims of the study [52]. Process models such as the knowledge to action framework can specify the stages to guide the process of translating research into practice [53]. The Promoting Action on Research Implementation in Health Service (PARIHS) framework [54], theory of diffusion [55], and the Capability, Opportunity, Motivation, and Behaviour (COM-B) model [56] are all examples of understanding what influences implementation outcomes, where the Reach, Efficacy, Adoption, Implementation, and Maintenance (RE-AIM) model focuses on examining the implementation strategy success [57]. Once an overall theory is established, implementation strategies can then be tailored to the trial organisation or individuals, as studies have shown tailored strategies may be more effective than generic strategies [58, 59].

It is worth noting the consideration of system levels when planning a counterbalanced study. By replicating a counterbalanced study across multiple systems (e.g. hospitals, healthcare organisations, public/private, primary/tertiary, states), more generalisable findings regarding implementation strategies applied in different contexts could be developed. Developing evidence in this way creates an opportunity to ensure external validity of findings, which would support the scale-up of implementation strategies [60]. Alternatively, it could be valuable to conduct a counterbalanced trial in a single system. Creating locally specific knowledge around the implementation process would re-direct the study focus to how strategies work within local organisational cultures. This would be useful for those interested in the internal validity of strategies and their applicability at a local site [61].

## Discussion

In this manuscript, we have described a novel approach to the evaluation of different implementation strategies across multiple health contexts. The counterbalanced implementation trial compares the same subject response to all interventions, effectively reducing the number of potential confounding covariates. This approach may improve the efficiency and precision of studies through smaller levels of variance, which can reduce the sample size required to identify a statistically significant change in outcomes [62]. In situations where implementation strategies cannot be evaluated concurrently, the potential for order effects needs to be considered. Homogeneity between implementation strategies or context areas would have to be addressed to avoid potential carryover or ‘learning’ effects, where the benefits of the implementation strategy in one context are potentially carried over to the next implementation strategy in the next context. Some implementation strategies are ‘health context specific’, in that they may be successful in one area but not in others [63, 64]. Researchers should consider whether their implementation strategy is transferrable across health contexts, or whether a counterbalanced design would be better employed to compare the strategy in the same health context across different organisations or study sites, rather than across contexts.

The applicability of traditional study designs to implementation science has been questioned [65]. Randomised controlled trials are important for determining the efficacy of treatments in highly controlled environments to ensure internal validity [66]. However, it has been suggested that these designs may not be appropriate for the implementation phase of evaluation, due to potentially low levels of external validity [65]. Novel designs, such as the counterbalanced, stepped-wedge [67], and adaptive trials [68] that incorporate the use of routinely collected health service data and incorporate consent waivers where ethically appropriate, may provide a logistically simple, low-cost pathway for the effectiveness and implementation phase of clinical research. Ideally, implementation evaluations would be conducted in ‘real-world’ settings, be appropriately statistically powered, and designed to reduce potential confounders and risk of bias that could mislead study conclusions [9]. Implementation studies often focus on the processes to integrate evidence-informed decision-making in healthcare organisations, involving clinicians, managers, and policy-makers [7]. These populations are notoriously time-poor [69–73], which can lead to difficulties in enrolling participants in an implementation study [17]. Therefore, study designs that can increase exposure to different conditions while maximising statistical power are valuable for implementation researchers. The counterbalanced implementation study design provides a pathway for progressive upscaling of successful implementation strategies in different health contexts, allowing gradual refinement of strategies for certain contexts. Upon study conclusion, all participants or participant clusters will have been provided with each implementation strategy, which can be continued if proved effective, cost-effective, and is taken up by the study organisation.

### Potential limitations of a counterbalanced design

Despite the advantages to conducting a counterbalanced implementation trial presented, there are limitations to applying this study design in some circumstances. The main limitations identified and described below relate to the feasibility and potential sources of bias.

#### Feasibility

A necessity of the counterbalanced implementation study is that recruited health services or individuals can be exposed to the target implementation strategies in each health context. Each individual participant or participating healthcare organisation would need to provide services towards each of the health contexts selected for the study. For example, if the two context areas were chosen, (1) reducing prescription of antibiotics for upper respiratory tract infections which have been shown to be overused [74] and (2) reducing the use of arthroscopic surgery for knee osteoarthritis which has no benefit for un-discriminated osteoarthritis [75], participants or participating healthcare organisations would need to be involved in both prescribing antibiotics for upper respiratory tract infections and routine treatment delivery for knee osteoarthritis. This may be difficult to achieve given the different disciplines and specialties involved.

The selection of certain health contexts and implementation strategies for evaluation may be restricted by the organisational policies, individual preferences, and limitations in resources provided at the health service or study location. Identifying multiple health contexts and implementation strategies in collaboration with organisations and individuals involved in a prospective counterbalanced implementation study may address this limitation. This collaborative approach may not differ largely from conventional implementation settings, where practice change must be aligned to organisational policies, goals, and priorities. An interesting approach that could account for these issues is ‘co-production’ or ‘co-design’ in implementation strategy development [76, 77]. This concept involves the collaborative development of implementation strategies by both the producer and user stakeholders [78] and may assist researchers and organisations in evaluating the implementation of programmes, practices, or policies.

Selection of outcomes in a counterbalanced implementation study also requires a consideration of feasibility, as specific measurements may differ between health contexts. We recommend patient or health service outcomes be used as standard measurements of implementation success where feasible. Feasibility should be determined prior to study conduct by considering the difficulty and cost of obtaining outcomes. Data such as hospital length of stay and rate of adverse events are routinely collected by health services and could therefore be considered examples of feasible outcome measures [9, 79, 80]. It must also be considered whether participant recruitment is likely to reach adequate thresholds as to effect outcomes within clusters (e.g. wards, hospitals). Alternatively, changes in attitudes, knowledge, behaviour, or implementation process outcomes may be considered, where outcomes are not based on routinely collected data (e.g. patient comprehension errors), or recruitment is unlikely to alter outcomes at the cluster level.

#### Potential source of bias

Two potential sources of bias in a counterbalanced implementation study are outcome and participant selection bias. Outcomes should be carefully selected and interpreted from a grounded theoretical basis, and pre-specified in clinical trial registration and published protocols to avoid the selection of ‘convenient’ outcomes, or selective reporting in published manuscripts. Participant selection bias can occur through low response rates in recruitment for counterbalanced implementation studies. Therefore, reporting should address whether there was a low response rate in participant recruitment and approaches used to address this potential risk of bias. In addition, there should be clear reporting when there are limitations to the application of results outside of the study sample.

### Future research

There are currently no reporting guidelines specific for counterbalanced randomised studies. Until consensus is established for the minimum standards for transparent reporting of counterbalanced trials, we recommend that reporting should follow the CONsolidated Standards of Reporting Trials (CONSORT) Statement 2010 [45, 81]. In addition, reporting of any testing (or non-testing) for carry-over effects of interventions across periods, and reporting of washout periods, should be included if clusters are exposed to study conditions sequentially. Other manuscript requirements from the International Committee of Medical Journal Editors (ICMJE) should also be considered [82]. Future research should focus on consensus reporting guidelines and analyses of counterbalanced implementation trials to ensure quality of conduct and reporting.

## Conclusion

The proposed novel counterbalanced implementation trial provides a potentially efficient and pragmatic research implementation study design for the evaluation of different strategies across multiple contexts. This design extends conventional trials used in the evaluation of implementation strategies. In the example application, comparing ‘video-’ versus ‘written-based’ research evidence summaries in community-acquired pneumonia and nutrition for critically ill patients, the counterbalanced implementation design would offer a potentially feasible and cost-effective means for evaluation and tailoring of implementation strategies in a study setting. The improved study balance through repeated measure design may result in proportionally fewer participants needed for adequate statistical power compared to potentially less balanced parallel approaches, allowing a ‘two for one’ evaluation of implementation strategies for high priority implementation contexts. Further refinement and tailoring of strategies within studies may facilitate scale-up of successful implementation strategies for translating research into practice across healthcare organisations.

## Additional files

Additional file 1: Terminology guide. (DOCX 46 kb)

Additional file 2: Main analysis model. (DOCX 68 kb)

Additional file 3: Interaction effects analysis model. (DOCX 79 kb)

## Abbreviations

CONSORT: CONsolidated Standards of Reporting Trials
ICMJE: International Committee of Medical Journal Editors
