# Supplementary material for: A novel counterbalanced implementation study design: methodological description and application to implementation research
Source: Implement Sci. 2019 May 2;14:45. doi: 10.1186/s13012-019-0896-0 (PMC6498461; doi:10.1186/s13012-019-0896-0)
Supplement: Supplementary file 1 — Terminology guide. (DOCX 46 kb) [file 13012_2019_896_MOESM1_ESM.docx]

| Terminology guide:  Cluster = unit of randomisation (e.g. hospital ward)  Intervention = implementation strategy (e.g. knowledge broker)  Context = condition, treatment, or setting (e.g. falls prevention)  Cluster Implementation intervention  “**Hospital wards** are randomised to receive **a knowledge broker or usual care** to increase **use of evidence-based strategies for the prevention of falls**”  Context |
| --- |
