# Supplementary material for: A novel counterbalanced implementation study design: methodological description and application to implementation research
Source: Implement Sci. 2019 May 2;14:45. doi: 10.1186/s13012-019-0896-0 (PMC6498461; doi:10.1186/s13012-019-0896-0)
Supplement: Supplementary file 2 — Main analysis model. (DOCX 68 kb) [file 13012_2019_896_MOESM2_ESM.docx]

| Y_uic_ = α + (β_1_ x_1ui_ + … + β_n_ x_nui_ ) + (γ_1_ z_1uc_ + … + γ_n_ z_muc_ ) + b_uic_ + a_u_…  Y = outcome for a unit of randomisation given a strategy/context combination  _u_ = unit of randomisation (cluster)  _i_ = implementation strategy (intervention)  _c_ = health context  α = overall intercept  β = fixed slope for implementation strategy  x = indicator variable of which implementation strategy is active  _n_ = number of implementation strategy levels  γ = fixed slope for health context  z = indicator variable of which health context is active  _m_ = number of health contexts  b_uic_ = residual  a_u_ = unit of randomisation specific deviation from the overall intercept | |
| --- | --- |

Where Y_uic_ is the outcome of each unit of randomisation to a given combination of implementation strategy and health context, (β_1_ x_1ui_ + … + β_n_ x_nui_ ) refers to which of _n_ strategies, and (γ_1_ z_1uc_ + … + γ_n_ z_muc_ ) refers to which of _m_ contexts.
